# Validity of the cohort of Crete in the Seven Countries Study: A time-series study applied to the cancer mortality trend between 1960 and 2011

**DOI:** 10.3892/ol.2013.1133

**Published:** 2013-01-14

**Authors:** CHRISTOS M. HATZIS, DIMITRA SIFAKI-PISTOLLA, CHRISTOPHER PAPANDREOU, GREGORY I. CHLOUVERAKIS, ANTHONY G. KAFATOS, NIKOLAOS E. TZANAKIS

**Affiliations:** 1 Department of Social Medicine, Preventive Medicine and Nutrition Clinic; Medical School of the University of Crete, Heraklion, Greece; 2 Laboratory of Biostatistics, Department of Social Medicine; Medical School of the University of Crete, Heraklion, Greece; 3 Department of Thoracic Medicine, University General Hospital; Medical School of the University of Crete, Heraklion, Greece; 4 Department of Epidemiology, Social Medicine, Medical School of the University of Crete, Heraklion, Greece

**Keywords:** cancer mortality, Seven Countries Study, time-series, rescaled range analysis, state-space reconstruction

## Abstract

We examined whether the Cretan cohort of the Seven Countries Study (SCS) is representative for the entire population in the island using cancer mortality registries. The analysis was carried out on the Cretan cohort of the SCS cancer mortality data and a similar cancer registry for the general population during a 51-year follow-up (1960–2011). Information about the causes of mortality was obtained from official death certificates and classified according to the International Classification of Diseases, 9th Revision (ICD-9). Two time-series models of mortalities from cancer, using data from the Cretan cohort and the Hellenic Statistical Office (EL. STAT), were developed using Matlab software. The existence of long-term memory in the data was tested by rescaled range analysis (Hurst-Mandelbrot). State-space reconstruction was applied to identify the simplest system that was able to re-create the present time-series. In the cohort, cancer mortalities accounted for 18.9% of total mortalities. The EL.STAT time-series analysis generated mean V statistics (95%CI) of 0.69815 (0.398–0.999) and 0.677143 (0.301–0.897) for the general population and the seven countries cohort, respectively. The embedding dimension for the EL.STAT data was equal to 1 for the general population and for the Cretan cohort (m=1). The exponent H values for the two time-series were almost equal. In the two time-series the proposed time delay of cancer mortalities was 2. The Cretan cohort of the SCS and the entire population of the island followed similar patterns of cancer mortality over time.

## Introduction

One of the challenges facing prospective research designs is the recruitment of large and representative samples of participants in order to enhance the external validity of the results. The majority of the larger famous cohorts assessing risk factors have widely been criticized as suffering from a non-representative studied subpopulation leading to marked sampling bias. For instance, the Framingham Heart Study, a cohort cardiovascular study on residents of the town of Framingham Massachusetts, has received extensive criticism concerning the representativeness of the study population. Researchers in particular wondered how closely the participants in the Framingham study resemble the ordinary patients who healthcare professionals meet in every-day practice (1). Another large cohort study, the Nurses’ Health Study, has followed 121,700 female registered nurses since the mid-1970s. The aim was to assess risk factors for cancer and cardiovascular disease. Due to the selected subpopulation (nurses), this study has been considered not representative of the general population with respect to socio-demographic factors and health care access (2). The Seven Countries Study (SCS) revealed that the population of Crete had the lowest rates of cardiovascular disease and cancer among 16 populations from seven countries (Greece, Finland, Japan, former Yugoslavia, Italy, the Netherlands and the United States) (3) and this was attributed to certain lifestyle and dietary factors. The findings of the above study contribute largely to the globally accepted statement that the Cretan diet, as a rigorous branch of the Mediterranean diet, is a remarkable determinant of the observed lower cardiovascular and cancer mortality. However, others have criticized the SCS as suffering from sampling bias (4). In particular, the major comment concerning the Cretan cohort is that its population was recruited from a specific rural area of Crete and could not be representative of the whole of Crete. Thus it is important to investigate those doubts.

An indirect method of investigating a representation of a subpopulation is to compare the temporal trends of cancer mortality rates of such a subgroup of a population with the figures of the general population from which it originated (5). Temporal trends in major cancer mortality causes predominantly reflect historical patterns of similar risk factors within a population (6). We hypothesized that this may be particularly true in a closed population such as that in Crete. Thus the time-trends of mortality from major cancer causes in the general Cretan population should present a similar pattern to the figures of a specified geographical region. Taking the above into account, the primary aim of this study was to examine whether the Cretan cohort is reliable and representative of the rest of the population in the island by comparing secular trends of major cancer mortality figures. A second aim was to compare the variance of cancer mortality rates of the SCS Cretan cohort during 51 years of follow-up with those of the general population of Crete during the same period.

## Materials and methods

### Sampling

The Cretan cohort of the SCS was identified in 1960 among 97.6% of all males aged between 40 and 59 years in 11 villages in the central region of Crete, Greece (3). The total sample included 686 participants. Follow-up examinations of the cohort took place 5, 10, 31, 37, 40 and 50 years after the baseline examinations. During the 50 years of follow-up, information about all causes of mortality was obtained from official death certificates. For the purpose of this study we extended the period for obtaining the information until 07/04/2011. The final adjudication of the underlying cause of mortality and the contributory causes was made according to the criteria of the World Health Organization (WHO) International Classification of Diseases, 9th Revision (ICD-9), by an expert medical epidemiologist. This study was approved by the ethical committee of the University of Crete.

### Statistical analysis

First, the number of mortalities from all types of cancer in Crete per year, as classified by the ICD-9 mortality classification system, was selected from the database of the Hellenic Statistical Authority (EL.STAT) between 1960 and 2011. The same was carried out for the mortalities from the Cretan cohort. These two sets of data were transformed into number of mortalities per 1,000 people. Secondly, the two sets of data were used throughout the whole analysis process. Time-series models were applied using Matlab. The two time-series were studied separately to estimate if they present the same behavior through time; behavior refers to their increases or decreases, variance, intensity and pattern of occurrence (1–4,7–20).

### Time-series analysis

Rescaled range analysis (Hurst-Mandelbrot) using a time-series filter of the first differences method was applied in order to examine whether there was long-term memory in our data (1–4,7,8). This method measures the H exponent. If H=0.5 then the time series follows the model of random walk (random memory). If 0<H≤0.5 then the time series would have an anti-persistence (with zero spectral density at the origin). Finally, if 0.5<H≤1 it means that there is a long-term memory. The V statistic was also measured by the same process to examine whether the time-series is deterministic or not.

Continuously, state-space reconstruction was applied. This refers to the method used to export conclusions regarding the simplest system that is able to re-create the present time-series (respectively), based on an observed quantity. We determined this to be the simplest system of the minimum possible number of variables that is able to reproduce this time-series. Space reconstruction is fulfilled by creating points, x_i_ɛR^m^ from single dimensional observations {x_i_} for i=1,…,N. The parameters of reconstruction are: embedding dimension ‘m’ that determines the number of observations which become components of the reconstructed vector; and delay ‘r’ which determines the time difference between selections of the parameters ‘m’ (8–14). The method of false nearest neighbors was applied in order to estimate the embedding dimension, and the Mutual Average Information method was used in order to carry out the time delay estimation (8–20). As a proposed time delay, we selected the one that was the first local minimum. The optimum time delay is one that will enable the data to be independent of one another when they are set as vectors with independent factors in an ‘m’-dimensional system of coordinates. This refers to independent data which are free of any kind of dependence. The time delay is presented for each I(r) in bits. This is the first local minimum of ‘r’ which presents the mutual average information (8–14).

## Results

The numbers and percentages of mortalities in the Cretan cohort and in Crete per type of cancer between 1960 and 2011 are indicated in Table I. In the cohort the total number of mortalities was 661, of which 125 were cancer deaths (18.9%). In Crete the number of cancer deaths was 3881 (∼24% of all causes of mortality) between 1960 and 2011. The first cause of cancer mortality was the gastrointestinal system accounting for one-third of cancer mortalities in the two populations. In the cohort, this was followed by cancer of the urinary system (26.4%), respiratory system (25.6%) and hematopoietic system (7.2%), whereas in the general population the cancer causes differ: cancer of the hematopoietic system (22.8%), respiratory system (18.3%) and urinary system (17.8%).

**Figure 1:**
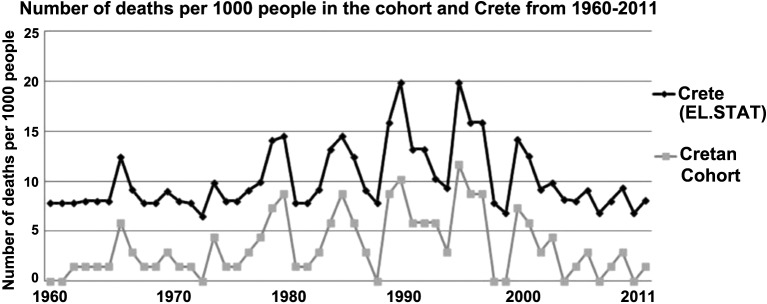
Numbers of mortalities in the Cretan cohort and Crete between 1960 and 2011.

Cancer deaths per 1,000 individuals between 1960 and 2011 are shown in Fig. 1, for the two sets of data. A greater number of cancer mortalities occurred in Crete but the root in time is equal to that in the Cretan cohort. In both cases, there are peaks in 1989 (8.75 deaths per 1,000 people in the Cretan cohort and 15.9 in Crete as a whole) and 1995 (11.66 deaths per 1,000 people in the Cretan cohort and 19.9 in Crete) since they increase or decrease through time in the exact same way. Finally, both populations present lower mortality rates in 1972 (1.46 deaths per 1,000 people in the Cretan cohort and 7.8 in Crete) and 1988 (0.02 deaths per 1,000 people in the Cretan cohort and 7.8 in Crete).

In Table II, the results of the rescaled range analysis are presented. The mean V statistic of the EL.STAT time-series was equal to 0.69815, at a confidence level of 95% (0.398–0.999) and that of the Cretan cohort’s database was equal to 0.677143, at a confidence level of 95% (0.301–0.897). Long-term memory existence was identified by calculation of the Exponent H (0.5<H≤1; Table II). Cancer mortality in the Cretan cohort and the whole island of Crete was found to follow the same non-random certain pattern through time since the exponent H values for the two time-series were almost equal (Table II).

The embedding dimension is presented through the method of false nearest neighbors in Table II. The embedding dimension for EL.STAT’s data was equal to 1, as was that for the Cretan cohort’s data (m=1). The time delay for this estimation (m=1) was equal to 1, a strong indication of a certain similarity between the nature of the two data sets.

Since only one parameter (variable) is needed for the cancer mortality system, it is important to identify the time period at which cancer mortalities occur at a significant level. The proposed time delay for cancer mortalities in the whole island of Crete was 2 and this was equal to the final time delay of cancer mortalities of the Cretan cohort (Tables III and IV). Therefore, the reconstruction system for the two time series was demonstrated to be exactly equal. Specifically, only one parameter and a two-year period should be used in order to recreate these two systems of cancer deaths in the future, in order to have correct, significant and reliable results.

## Discussion

Our study examined for the first time the representativeness of the SCS Cretan cohort by investigating cancer mortality rates over a 50-year period. The main findings of our results showed a strong tendency for the Cretan cohort and the entire island population to be moving toward similar cancer mortality patterns.

Specifically, the Cretan cohort included a sample of 686 males aged between 40 and 59 years old, which has been often criticized about its reliability (4). Although the SCS cohort exhibited a lower cancer incidence than the general Cretan population, our data indicate that the cancer mortalities of the two populations showed a similar pattern concerning cancer mortality over time. Additionally, a significantly larger number (than the mean number of mortalities of the previous years) of cancer deaths in the Cretan cohort occurred every 2 years as in the Cretan general population. Thus, the rate of cancer mortality occurrence is expected to be exactly the same for the Cretan cohort and Crete as a whole, having similar increases or decreases (behavior). The two time-series were constructed from the same number of factors. Consequently, the cohort could be generalized to the total population of Crete with regard to cancers, with no fear of mis-estimations (12,13,20–24). Similar to our observation, a USA study that examined cancer mortality trends between 1950 and 1969 in New Jersey, New York and Philadelphia in comparison with the remainder of the country also revealed a strong tendency for the region and the remainder of the nation to be moving towards similar mortality patterns (6). Another study conducted by Meslé *et al* was the first step in an exploration of the Soviet cause of death statistics which became accessible after 1986. Its aim was the reconstruction of consistent annual series for the period 1970–1987. Similar to our study, cancer mortalities presented increasing trends for the whole nation, particularly among females (25). In addition, the study by Anderson used interrupted time-series models and revealed that cancer mortality among males in eastern New England was increasing at a rate consistently higher than and almost parallel to national male cancer mortality, while female cancer mortality was declining, but less rapidly than nationally (26). Other studies have been carried out using similar methods of analysis on various causes of mortality, testing different parameters than those in our study. Stroup *et al* applied multiple time-series analysis to estimate the impact of influenza on mortality in various age groups, using a procedure for updating estimates as current data become available from national mortality data collected between 1962 and 1983. The study found differences among genders and time points (27). It is clear that in epidemiology, data often arise in the form of time-series, for example, notifications of diseases, entries to a hospital and mortality rates. Time-series models (especially, non-linear ones) are usually applied to such data, as in several studies, including the present one (6,14,23,24).

Cancer is a leading cause of mortality worldwide and accounted for around 13% of all deaths in 2008 (28). The present study revealed that nearly one-fifth of males from the Cretan cohort succumbed to cancer during a 51-year period and one-quarter of males from Crete as a whole during the same period. Similar observations were made in a 40-year follow-up study in the US Railroad cohort of the SCS, which indicated that cancer accounted for 25.1% of all causes of mortality (29). Cancer of the gastrointestinal system was the main cause of cancer deaths in both Cretan populations, followed by cancer of the urinary and respiratory system. Cancer studies of certain years (2004, 2006 and 2008) in Greece and Europe revealed that lung cancer was the main cause of mortality from cancer among males (30–32). The same trend was observed worldwide according to data for the year 2008 (33). However, conclusions on cancer mortality should not only be influenced by our present knowledge but also from its own ‘nature’. Time-series models aim at this goal: a complete description of the nature of cancer mortality (14,21).

In conclusion, the present study revealed that the two time-series for deaths from cancer (Cretan cohort and EL.STAT) were of the same behavior, pattern, nature and reconstruction over time, with the Cretan cohort having a slightly lower number of mortalities per year. The two time-series present similar results. These observations support the statement that the Seven Countries’ Study Cretan cohort is representative of the island’s entire population.

## Figures and Tables

**Table I:** Number of cancer mortalities in the cohort and in Crete per type of cancer among males, between 1960 and 2011.

Cause of mortality (type of cancer)	No. of mortalities in the cohort (%)	No. of mortalities in Crete (%)
Respiratory system	32 (25.6)	712 (18.3)
Urinary system	33 (26.4)	694 (17.9)
Gastrointestinal system	38 (30.4)	1183 (30.5)
Hematopoietic system	9 (7.2)	886 (22.8)
Brain	5 (4.0)	44 (1.1)
Other	8 (6.4)	362 (9.3)
Total	125 (100)	3881 (100)

**Table II:** Rescaled range analysis and embedding dimension applied to the time-series of the Cretan cohort and Crete.

	Cretan cohort time-series	Crete (EL.STAT) time-series
Mean V statistic	0.677143	0.69815
Exponent H (min; max)	0.286138; 0.514697	0.322975; 0.534748
Embedding dimension	1	1
Time delay	1	

**Table III:** Estimation of the proposed time delay applied on the number of cancer mortalities between 1961 and 2011 in Crete, using data from the EL.STAT database.

Final time delay	I(r)	Final time delay	I(r)
0	1	14	−0.1439
1	0.3814	15	−0.06178
2	0.02021	16	0.05236
3	−0.02785	17	−0.05082
4	0.1095	18	−0.1592
5	0.5218	19	−0.2324
6	0.3641	20	−0.1837
7	0.02206	21	−0.07677
8	−0.1162	22	−0.1271
9	−0.1503	23	−0.07435
10	0.2171	24	−0.1503
11	0.2393	25	−0.1827
12	−0.00186	26	−0.11
13	−0.1439		

Proposed time delay: 2.

**Table IV:** Estimation of the proposed time delay applied on the number of cancer mortalities between 1961 and 2011 in Crete, using data from the Cretan cohort database.

Final time delay	I(r)	Final time delay	I(r)
0	1	14	−0.2642
1	0.4178	15	−0.14
2	0.06165	16	0.002099
3	0.04649	17	−0.04582
4	0.1928	18	−0.2015
5	0.4276	19	−0.2615
6	0.4036	20	−0.2318
7	0.0521	21	−0.1406
8	−0.07681	22	−0.1558
9	−0.1375	23	−0.0567
10	0.1524	24	−0.2072
11	0.2367	25	−0.2215
12	0.04178	26	−0.1127
13	−0.1232		

Proposed time delay: 2.
